# Organic Contaminant-Triggered Self-Healing Soil Mix Cut-Off Wall Materials Incorporating Oil Sorbents

**DOI:** 10.3390/ma13245802

**Published:** 2020-12-18

**Authors:** Benyi Cao, Livia Ribeiro de Souza, Abir Al-Tabbaa

**Affiliations:** Department of Engineering, University of Cambridge, Trumpington Street, Cambridge CB2 1PZ, UK; lrds2@cam.ac.uk (L.R.d.S.); aa22@cam.ac.uk (A.A.-T.)

**Keywords:** self-healing, cut-off wall, organic contaminant, oil sorbent

## Abstract

Soil mix cut-off walls have been increasingly used for containment of organic contaminants in polluted land. However, the mixed soil is susceptible to deterioration due to aggressive environmental and mechanical stresses, leading to crack-originated damage and requiring costly maintenance. This paper proposed a novel approach to achieve self-healing properties of soil mix cut-off wall materials triggered by the ingress of organic contaminants. Oil sorbent polymers with high absorption and swelling capacities were incorporated in a cementitious grout and mixed with soil using a laboratory-scale auger setup. The self-healing performance results showed that 500 µm-wide cracks could be bridged and blocked by the swollen oil sorbents, and that the permeability was reduced by almost an order of magnitude after the permeation of liquid paraffin. It was shown by micro-CT scan tests that the network formed by the swollen oil sorbents acted as attachments and binder, preventing the cracked mixed soil sample from crumbling, and that the oil sorbents swelled three times in volume and therefore occupied the air space and blocked the cracks in the matrix. These promising results exhibit the potential for the oil sorbents to provide soil mix cut-off walls in organically-contaminated land with self-healing properties and enhanced durability.

## 1. Introduction

The problem of soil contamination resulting from industrial waste storage, treatment and disposal practices, and from accidental spills, is of widespread concern. Among the most common soil contamination sources are former manufactured petrol plants, and as a result, a lot of potentially fertile soils were contaminated with organic contaminants [1,2]. Present technologies for remedial action often involve disruption of the contaminant transport pathway from a contaminant source zone to a receptor [3]. Although soil mixing was developed in the 1960s in Japan and Sweden for geotechnical applications, its potential in geo-environmental applications became evident in the 1980s in the USA and in the 1990s in Europe for the remediation of contaminated land [4]. Soil mixing auger systems create columns as the soil is mixed, and by using a self-hardening cementitious grout mixture and by overlapping columns, low permeability cut-off walls can be constructed. Soil mix cut-off walls have successfully been employed in organically contaminated sites as a containment technology, such as the Sir John Rogerson’s Quay Project in Dublin, Ireland [5], and Project SMiRT in Castleford, UK [4].

Despite the successful commercial applications of soil mix cut-off walls in polluted land, the inevitable crack-originated damage and chemical degradation can undermine their integrity and serviceability. Although cut-off walls are not designed to transmit load, it is common for these walls to experience changes in loading conditions due to subsequent construction activities associated with redevelopment, which can cause deformation and cracking [6]. Environmental stressors such as wet-dry cycles can also contribute to the cracking of soil mix cut-off walls, resulting in significant increase in permeability by up to six orders of magnitude [7]. A major concern with such damage of in-ground cut-off walls is that it is usually very difficult to inspect and repair [8], making the concept of self-healing cut-off wall materials extremely attractive.

Self-healing in cementitious materials is triggered by physical or chemical factors. Microcapsule-based self-healing, for example, is often triggered by tensile stress in the matrix, leading to the rupture of microcapsule shell and release of healing agents [9,10,11]. Polymer-based self-healing approaches, on the other hand, are usually chemically triggered. Extensive studies incorporated superabsorbent polymers (SAPs) in cementitious materials, and found that the swelling of SAPs triggered by the ingress of water could effectively seal cracks [12,13,14]. Following the idea of SAP-based self-healing, oil absorbent polymers, also known as oil sorbents, have recently been studied for the development of self-healing oil well cement systems [15]. When hydrocarbons come in contact with oil sorbents, the particles swell, enabling the cement sheath to have self-healing properties. To achieve a high absorption capacity, desired properties of oil sorbents include: (1) good mutual solubility, which is governed by the chemical structure—the chain structure of both organic liquids and absorbents should be similar; (2) high surface area, which maximizes the sorption surface available; (3) porous structure, which further facilitates the migration of organic liquids into the structure of absorbents; (4) a fairly low degree of cross-linking, which improves the swelling capacity of a polymer structure and hence improves its absorption capacity [16]. The reported absorption capacity of oil sorbents varied from 5 to 140 g/g in different organic liquids, with the typical values ranging from 5 to 40 g/g.

Lu et al. [17] synthesized a styrene-butyl acrylate copolymer with the absorptivity of 7 g/g in diesel and the size of ~180 nm. The self-healing performance of the cracks in the oil well cement specimens with and without oil absorbents was measured by the oil flow test under different pressures. According to the authors’ calculation, the width of the crack in the control sample remains almost unchanged during the curing in the oil bath for a period of 6 days, while the crack width in the oil sorbent-containing sample significantly decreases from 25 μm to 12 μm. Wang et al. [18] synthesized a methyl methacrylate-lauryl methacrylate copolymer with an average size of 207 µm and an absorption capacity of 5.5 g/g in kerosene. The larger size of oil sorbent particles enabled the cement samples to self-heal wider cracks up to 180 µm. More recently, the self-healing performance of oil sorbents in oil well cement were investigated by Zhang et al. [19]. After cracking, the cement samples were immersed in kerosene at 80 °C. The results showed that when the oil sorbent dosage increased from 0% to 8.0%, permeability decreased from 2.64 × 10^−3^ to 0.25 × 10^−3^ μm^2^ after 30 days of healing. The first commercial deployment of oil sorbents in oil cement systems was conducted by Schlumberger to prevent leaks in the Berliner Gaswerke AG, and it was observed that the gas flow rate was reduced by up to 100% [20].

Despite the development of self-healing oil well cement, the incorporation of oil sorbents in geotechnical infrastructure, including soil mix cut-off walls, has not been investigated. The concept of chemically-triggered self-healing oil sorbent-based soil mix cut-off wall materials is explored for the first time in this paper: oil sorbents are incorporated in the cementitious grout and mixed with the in-situ soil using an auger mixing system, and the oil sorbents can swell and then block cracks when exposed to the ingress of organic contaminants. The objectives of this study are to: (1) demonstrate the feasibility of soil mixing with oil sorbent-incorporated cementitious grout using a laboratory-scale auger setup; (2) investigate the effects of oil sorbents on the properties of the grout and mixed soil columns; (3) demonstrate the swelling behavior of oil sorbents in the mixed soil, triggered by organic contaminants, and the self-healing performance in terms of crack blockage and recovered low permeability; and (4) explore the self-healing process and mechanism with microstructural analyses including SEM-EDX and micro-CT scan.

## 2. Materials and Methods

### 2.1. Materials

A sharp sand provided by Ridgeons, Llangefni, UK was used as a model sand soil to replicate the particle size range of the sand stratum at the made ground of land remediation sites. The sand used had particle sizes ranging from 0.07 to 1.18 mm with a median particle size of 0.36 mm. Potable water was used to maintain the water content of the soil at 20%. The soils were placed in tinplate buckets, 310 mm in diameter and 400 mm high, using consistent moderate compaction in four equal layers of 100 mm each. Grout binders used in this study consisted of CEM I 52.5 N Portland cement (PC) and ground granulated blast furnace slag (GGBS) supplied by Hanson, Cambridge, UK.

The oil sorbent, a styrene-ethylene/butylene-styrene (SEBS) polymer, was supplied by M^2^ Polymer Technologies, Dundee, IL, USA. This oil sorbent product is especially effective with volatile and high Kb-value solvents such as hexane, liquid paraffin, gasoline, diesel, kerosene, benzene, and chlorinated solvents. The product crosslinks to encapsulate organics and forms a rubbery mass that can seal the cracks. The oil sorbents were delivered in the form of white rubbery particles with a bulk density of ~350 kg/m^3^ and an average particle diameter of 520 ± 230 µm. In this study, liquid paraffin was chosen as the representative organic contaminant due to its good stability and low toxicity.

Three mixes were used in this study to investigate the self-healing performance of mixed soil incorporating varying dosages of oil sorbents. Grouts were prepared in a high-speed mixer by first dry mixing cement, GGBS, and oil sorbents and then adding and mixing pre-hydrated bentonite slurry (5% bentonite in water and hydrated for 24 h) with the binders together. A soil-to-grout ratio of 4:1 and a binder-to-water ratio of 1:1 were used. The ratios of constituents of the control and oil sorbent-containing mixes are detailed in Table 1. The percentages at the end of the mix identification indicate the mass fraction of oil sorbents with respect to the grout weight.

### 2.2. Laboratory-Scale Auger Setup

**Figure 1:**
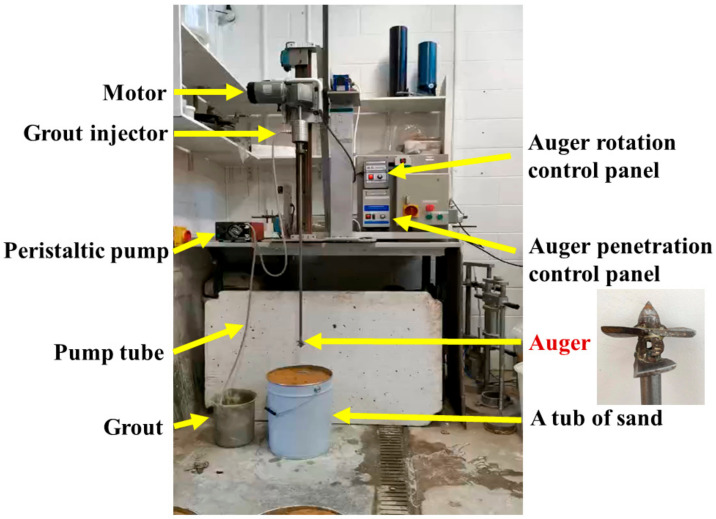
Laboratory-scale auger setup used in this study for the formation of soil-grout columns.

A laboratory-scale auger setup, as shown in Figure 1, was used to produce mixed soil columns. The automated system consisted of a vertical track that enables the penetration and retreat of the auger and grout injector component. Grout is pumped into the grout injector from a peristaltic flow pump down the hollow shaft section of the auger. The auger head used in this study had two layers of blades with sharpened edges and a blade axis twist of 5°. The cutter diameter of 50 mm was used to produce samples suitable for strength and permeability tests.

The auger penetrated the soil at a speed of 5 mm/s in a clockwise direction with a rotation speed of 50 rpm and to the required depth of 300 mm. Injection of the grout then took place on the retreat of the auger, while the direction of rotation was reversed. After injecting the full requisite quantity of grout, two further mixing cycles were performed without further grout injection to ensure the effective mixing of the soil and the grout. After 7 days of mixing, all the mixed columns were extruded, and trimmed using an electrical saw into 100 mm long samples to be tested for mechanical and permeability properties. The extruded columns were placed in a curing incubator until the day of testing. All specimens were mixed in a standard laboratory environment of 21 °C (±2 °C) and 60% (±10%) relative humidity (RH) and cured at 21 °C (±2 °C) and 98% (±2%) RH.

### 2.3. Experimental Methods

The filtration method was used to measure the absorption characteristics of the oil sorbents. Organic fluids were added to 1.0 g dry oil sorbent particles and the whole was filtered after one day. The absorption capacity was calculated from the weight increase between the dry state and the saturated state.

The unconfined compressive strength (UCS) was obtained using the trimmed cylindrical samples tested in a 50 kN servohydraulic compression frame. The vertical load was applied axially at a constant rate of strain of 1 mm/min until failure, from which the strength was then calculated. In addition, a linear variable differential transformer was used to measure the vertical displacement of the specimen in order to calculate the axial strain and the secant modulus of elasticity (*E*_50_). The secant modulus was calculated by measuring the slope of the straight line drawn between the zero point and the stress at 50% of the maximum stress at failure.

The vertical permeability of the specimens was determined by a constant flow rate test using flexible wall permeameters and peristaltic flow pumps. A steady flow rate was applied at the bottom of the sample using flow pumps. A pore pressure transducer positioned at the inflow position measured the pore water pressure generated. When a constant pressure had been reached, the vertical permeability of the sample was determined. The samples were first tested after 28-day curing and then they were cracked using the UCS frame until the peak force was reached. The cracked samples were permeated with liquid paraffin for six hours and the recovered properties were tested to assess the self-healing efficacy.

The SEM was used to characterize the microstructural morphology of the specimens and EDX was used to study the elemental composition. Small chipped pieces were collected from the cracked specimens. Phenom ProX SEM (Thermo Fisher Scientific, Waltham, MA, USA) was used, and the specimens were scanned and images were captured at different magnifications.

Micro-CT was used to investigate the distribution and morphology of the oil sorbents before and after the absorption of liquid paraffin, as well as the blockage of cracks by the swollen oil sorbents. The X-ray CT scanner used was the Nikon XT H 225 ST CT scanning apparatus (Nikon Metrology, Derby, UK). In this study, a specimen with the size of ~15 mm × 15 mm × 15 mm was scanned. The first scan was carried out on the undamaged sample after 28-day curing; the sample was then cracked by the UCS frame and immersed in liquid paraffin for six hours and the second test was conducted. The reconstructed image matrix had a volume of 2000 × 2000 × 1000 and the effective voxel size was 5 µm. The density of each voxel, represented by the material local linear attenuation coefficient, was normalized to 16-bit grey values, and specialized rendering software (VGStudio MAX) (Volume Graphics, Heidelberg, Germany) allowed for visual inspection of this 3D volume.

## 3. Results and Discussion

### 3.1. Feasibility of Using Oil Sorbents for Self-Healing in Soil Mix Cut-Off Walls

**Figure 2:**
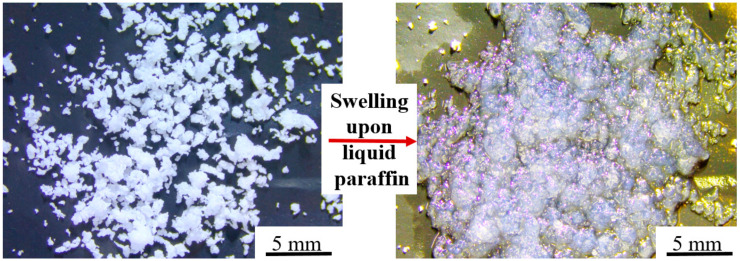
The swelling of oil sorbent particles after absorbing liquid paraffin.

The expansion of the oil sorbents triggered by organic contaminants plays an important role in healing the cracks in the soil mix cut-off walls. The volume expansion of the oil sorbents immersed in liquid paraffin for 10 min is shown in Figure 2. The filtration test results show that the oil sorbents have a very high absorption capacity for pure straight-chain hydrocarbons such as hexane, at up to 23 g/g. This is because hexane has a linear structure similar to the chemical structure of the oil sorbent polymers, making it easy for the hexane molecules to be absorbed to and enter the oil sorbents. The absorption capacity for medium-density refined oil products was 10–13 g/g including liquid paraffin, diesel, mineral oil, and benzene. These values are comparable to the ones found in the literature, such as Nam et al. [21], who synthesized an oil sorbent consisting of ethylene/1-octene copolymers with a diesel absorption capacity of 7.6 g/g. Zhang et al. [19] produced a lauryl methacrylate-styrene-hydroxyl ethyl methacrylate polymer that had an absorption capacity of 12.7 g/g in paraffin.

### 3.2. Effect of Oil Sorbent on Fresh Grout Properties

**Figure 3:**
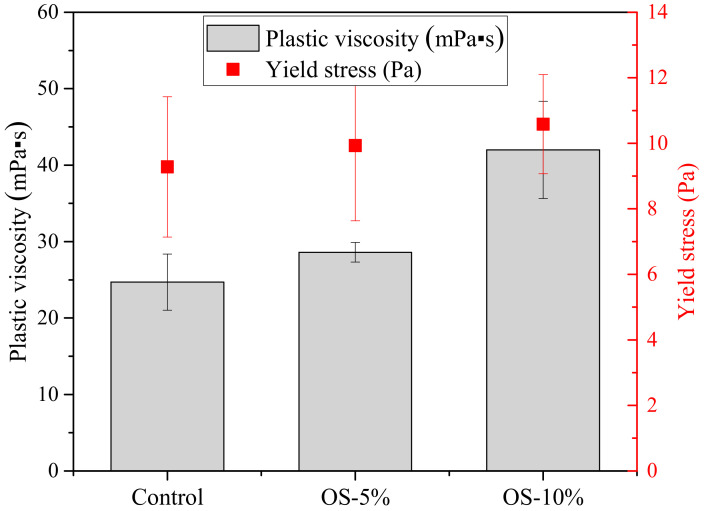
Plastic viscosity and yield stress of control and grouts with oil sorbents contents of 5% and 10%.

The proper rheological behavior of fresh grout plays an important role in the successful mixing of soil with the grout, as the pumpability of a grout mixture is primarily defined by its viscosity. The grout should be flowable enough to make sure it can be pumped through an auger without any blockage. Figure 3 shows that the plastic viscosity and yield stress presented an almost linear increase with the increasing oil sorbent content. The plastic viscosities of the grouts with the oil sorbent contents of 0%, 5% and 10% were 24, 28, and 42 mPa·s, respectively. The addition of oil sorbent at 10% increased the viscosity of grout by 75% compared with the control sample. As the flowability decreased, higher energy was needed to initiate the flow of the grout, thus increasing the yield stress. The yield stress with the oil sorbent contents of 5% and 10% grouts increased to 9.9 and 10.6 Pa from the control value of 9.2 Pa. Previous studies have also reported that the addition of polymers significantly increased the viscosity and reduced the fluidity of cementitious materials. It was found that the cement grout containing 5% epoxy resin appeared to have a plastic viscosity increase of 80% compared with the control sample [22]. To achieve proper workability of the cement grout, the plastic viscosity of the fresh grout should be less than 160 mPa·s [23], as it is the case for all the plastic viscosity values of the oil sorbent-containing grouts in this study. As a result, the addition of the oil sorbents at a dosage of up to 10% by grout weight is feasible, in terms of workability, for the construction of soil mix cut-off walls.

### 3.3. Physical and Mechanical Properties of Oil Sorbents for Self-Healing in Soil Mix Cut-Off Walls

**Figure 4:**
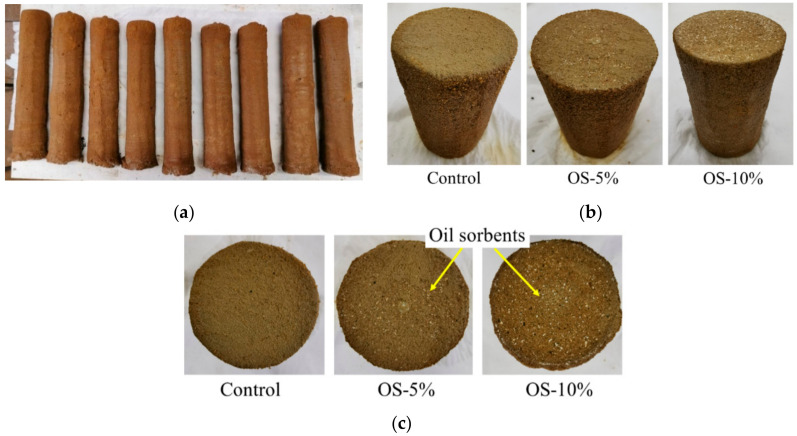
Representative images of (**a**) extruded mixed soil columns; (**b**) mixed soil samples after sawing and trimming; (**c**) cross-sections of the mixed soil samples.

Control and oil sorbent-containing mixed soil samples were extruded from the sand container after 7 days of auger mixing (Figure 4). The actual column lengths corresponded well with the intended lengths (300 mm) and the overall column diameters ranged from 55–60 mm. The diameters were 10–20% larger than the diameter of the auger, suggesting some spreading of the grout beyond the auger blade edges. Closer inspection after the sawing and trimming of the columns showed that the cylinder samples had smooth and homogenous surfaces on both ends and the side. In general, the columns had very good consistency for both diameters and length with no evident pockets of pure sand or binder within them, indicating the oil sorbents did not agglomerate or affect adversely the soil mixing process.

**Figure 5:**
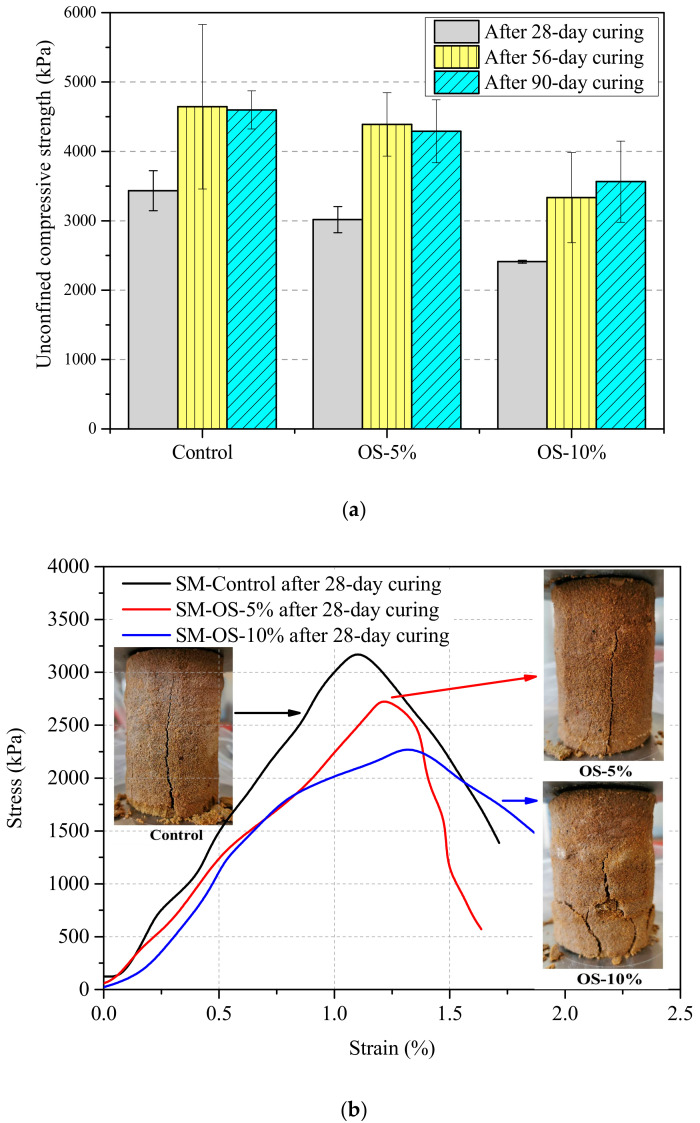
The effects of oil sorbents addition on: (**a**) unconfined compressive strength (UCS) at different ages; (**b**) stress-strain curves of control and oil sorbent-containing mixed soil samples.

The UCS of the mixed soil samples with and without oil sorbents were tested at 28, 56, and 90 days as shown in Figure 5a. The results show that the addition of the oil sorbents at a dosage of 5% and 10% generally decreased the UCS regardless of the age of the samples. After 28 days of curing, the UCS values of the control, OS-5%, and OS-10% samples were 3433, 3016, and 2410 kPa, respectively. With the increase of sample age, the adverse effect of oil sorbents on UCS did not become more significant. After 90-day curing, compared with the UCS of the control samples of 4598 kPa, the UCS values of OS-5% and OS-10% decreased by 7% and 22%, respectively. The oil sorbent particles are an elastomer and have low Young’s modulus and high failure strain compared with cement [24], so the oil sorbent particles could not resist or transmit load in the mixed soil matrix due to their low stiffness. The decreasing trends on UCS due to the oil sorbent addition agreed with previous research in the literature. Wang et al. [18] incorporated an oil absorbent polymer (methyl methacrylate-lauryl methacrylate) in oil well cement, and it was reported that the compressive strength was significantly decreased by about 28% and 41% with the addition of oil absorbent polymers at dosages of 5% and 8% by cement weight, respectively.

The representative stress-strain curves and crack patterns of the control and oil sorbent-containing samples are presented in Figure 5b. The cracks found on the surfaces of the control and oil sorbent-containing samples were mostly vertical, indicating the splitting failure pattern of the mixed soil was not affected by the addition of oil sorbents. The stress-strain behavior of oil sorbent-containing soil mix samples becomes more ductile as the oil sorbent content increases, despite the decreased peak stress with the addition of oil sorbents. The strains at failure of the OS-5% and OS-10% samples were 1.2% and 1.3%, compared to the control value of 1.1%. The values of *E*_50_ were 303, 248, and 221 MPa for the control, OS-5%, and OS-10% samples, suggesting the decreased stiffness was due to the incorporation of the highly deformable and rubbery oil sorbents. The reason is that the polymeric oil sorbents absorb a significant amount of strain energy and dissipate stress by deforming. For the oil sorbent-containing samples, large plastic deformations occur and much of the energy is consumed by the breakage of oil sorbents and dislocation glide of the polymer network structure. The increased ductility due to oil sorbent addition is beneficial to the soil mix cut-off wall materials because the cementitious matrix could deform to a greater extent without cracking, reducing the risk of the increase in permeability.

### 3.4. Blockage of Cracks

**Figure 6:**
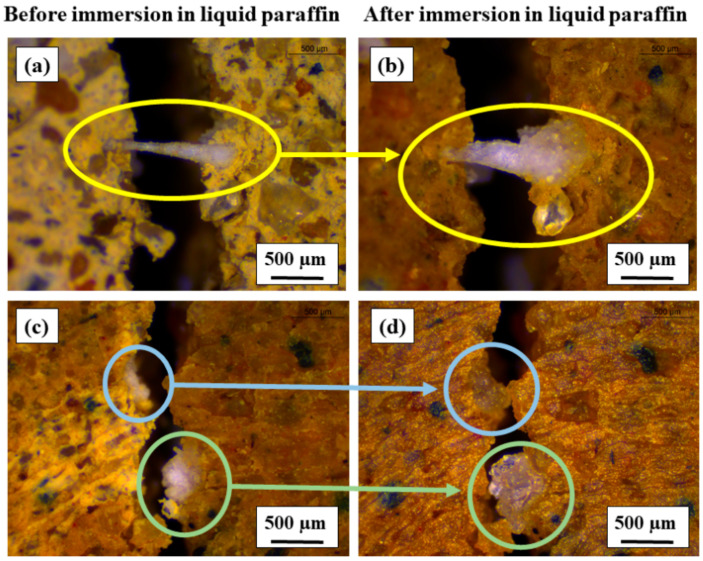
Blockage of cracks by liquid paraffin-triggered swollen oil sorbents: (**a**,**c**) oil sorbents before immersion and (**b**,**d**) oil sorbents after immersion in liquid paraffin.

The self-healing performance of the oil sorbent-containing samples is expected when the oil sorbents on the crack surface are exposed to organic contaminants. After 28 days of curing, an OS-10% sample was cracked under UCS test and then immersed in liquid paraffin for 30 min. Microscope images were taken using a Leica stereo microscope, and the visualization of crack blockage was conducted by comparing the images before and after the immersion in liquid paraffin. Two types of oil sorbents were observed on the cracked surface in Figure 6: (1) stretched oil sorbents anchored on both sides of the crack and (2) oil sorbent particles embedded in one side of the crack. Both types of the oil sorbents swelled significantly after the immersion in liquid paraffin. In Figure 6a,b, the area of the stretched oil sorbents increased to around 0.21 mm^2^ after the immersion compared to its original area of 0.13 mm^2^. The two-fold increase in the area of oil sorbents could make it more difficult for the permeated organic contaminants to flow through the crack. In Figure 6c,d, the area of the larger embedded oil sorbent particle increased from its original area of around 0.43 mm^2^ to 1.34 mm^2^. The considerable swelling of the oil sorbent particle managed to bridge and block a 500 µm-wide crack, indicating effective self-healing performance in terms of crack blockage. Improved crack blockage in oil well cement incorporating oil sorbents has been successfully demonstrated for 180 µm-wide cracks [18]. The maximum crack width that can be blocked is primarily controlled by the size and absorption capacity of the oil sorbent polymers. In general, larger oil sorbents with higher oil absorption capacity are able to block wider cracks. In Wang et al.’s research [18], the oil sorbents had an average particle size of 207 µm and a paraffin absorption capacity of 5.5 g/g. In comparison, the oil sorbents used in this study had an average particle size of 520 µm and a paraffin absorption capacity of 12.4 g/g, and therefore the healable crack width in this study was as much as 500 µm. The microscope images only presented an indication of the blockage at the cracking mouth but did not reflect the overall self-healing performance of the mixed soil samples. Therefore, investigations on the recovery of permeability as described in the following section were carried out to obtain more comprehensive information about the self-healing efficacy.

### 3.5. Recovery of Permeability in Soil Mix Cut-Off Walls Oil Sorbents

**Figure 7:**
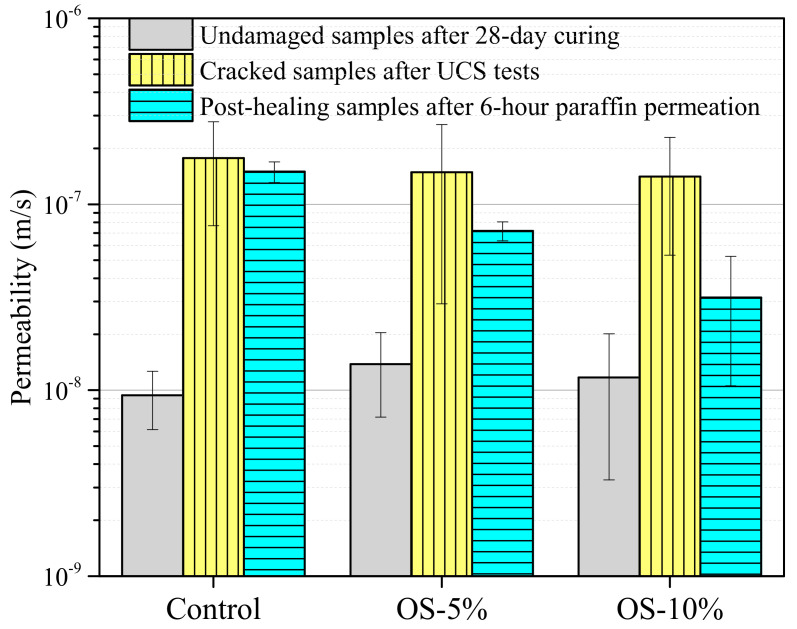
Permeabilities of undamaged, cracked, and post-healing control and oil sorbent-containing samples.

The permeability results of the undamaged, cracked, and post-healing mixed soil samples are shown in Figure 7. After the cracking using the UCS frame, the damaged control, OS-5%, and OS-10% samples had similar permeabilities of 1.7 × 10^−7^, 1.5 × 10^−7^, and 1.4 × 10^−7^ m/s, all of which were higher than the undamaged values (~1.0 × 10^−8^ m/s) by an order of magnitude. After the six-hour permeation of liquid paraffin, the oil sorbents on the crack surface swelled and blocked the crack volume, thus decreasing the permeability. In the post-healing samples, the permeabilities of OS-5% and OS-10% samples dropped to 7.2 × 10^−8^ and 3.1 × 10^−8^ m/s, compared to unchanged permeability of the control samples. Similar results of permeability recovery were obtained in previous studies on oil well cement incorporating oil sorbents. Zhang et al. [19] added a lauryl methacrylate-styrene-hydroxyl ethyl methacrylate copolymer with a paraffin absorption of 12.7 g/g in oil well cement, and they reported that the permeability decreased from 2.64 × 10^−3^ to 0.25 × 10^−3^ μm^2^ after the samples were immersed in paraffin for 30 days. The results also showed that the permeability of the post-healing samples decreased as the oil sorbent content increased. Adding 5% oil sorbents reduced the permeability by half of an order of magnitude, and a content of 10% substantially reduced the permeability by almost an order of magnitude. It is, however, not suggested that the oil sorbents be added at a very high content, because adding oil sorbents can adversely affect the strength of the mixed soil. It is therefore a trade-off between reducing the strength and improving the self-healing performance. The oil sorbent content of 10% used in this study could provide a reference: the post-healing permeability dropped by almost an order of magnitude, although the UCS decreased by 22%.

### 3.6. Morphology of Oil Sorbents in Soil Mix Cut-Off Walls

**Figure 8:**
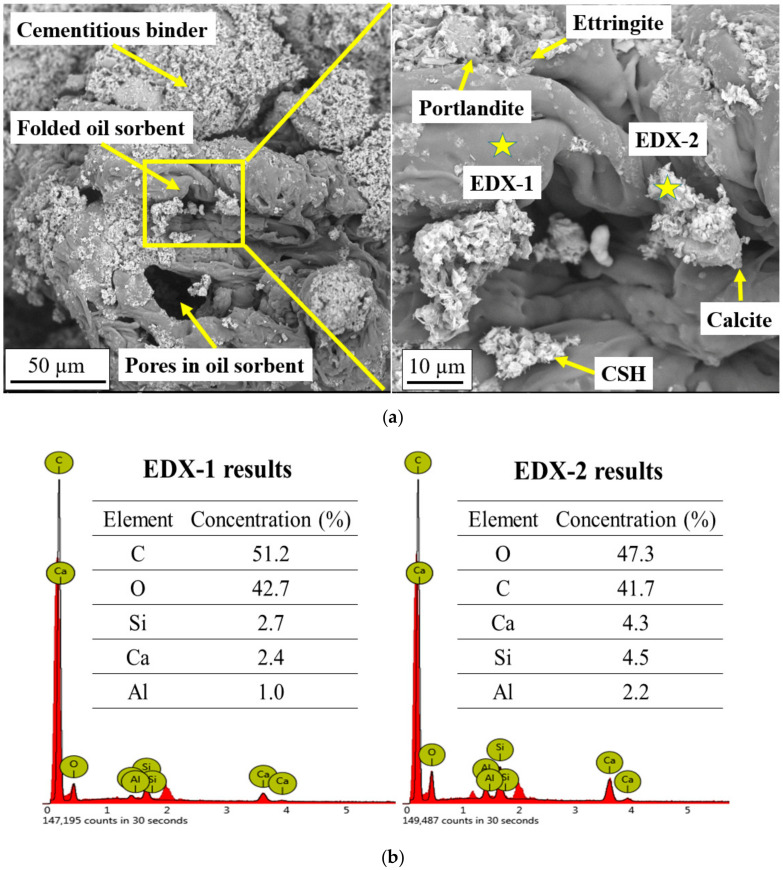
Representative results of (**a**) SEM image of an unswollen oil sorbent particle in cementitious matrix and (**b**) EDX analysis of two points on the oil sorbent.

To investigate the microstructure of mixed soil samples incorporating oil sorbents before and after the absorption of liquid paraffin, SEM-EDX analysis was conducted. Before the permeation of liquid paraffin, folded oil sorbent particles were observed on the cracking surface (Figure 8a). The oil sorbent particle firmly embedded itself into the cementitious matrix, indicating the strong bonding between the cement binder and the polymers. The bonding strength and friction between the polymer and the cementitious particles enabled the oil sorbents to bear tensile stress that developed during the cracking process, thus preventing the further opening of cracks. Macro pores with a size of ~30 µm could also be observed within the folded oil sorbent particles due to the porous structure of oil sorbents. However, these pores were not interconnected and did not extend through the entire particle, so the adverse effect of oil sorbents on the undamaged permeability was negligible. The hydrophobic hydrocarbon polymer backbone of styrene-ethylene/butylene-styrene polymer enables the adsorption of organic contaminants; however, it also hinders the chemical bonding with the cement hydration. Nonetheless, some cement hydration products were observed to precipitate on the surface and between the folds of the oil sorbent, likely due to weak van der Waals forces. These hydration products included clusters of fibrous calcium silicate hydrate (CSH), large hexagonal portlandite, needle-like ettringite, and cubic calcite.

To further analyze the oil sorbents and hydration products, EDX was used to determine their elemental composition. EDX analysis was carried out on two locations in Figure 8a. A variety of chemical elements, including oxygen, carbon, calcium, silicon, and aluminum, were detected on the seemingly smooth polymer surface (Figure 8b). This shows that the oil sorbent surface was covered with tiny cement hydration products. Likewise, the EDX-2 located on the cementitious particles also showed the presence of carbon, indicating that the particle was actually a mix of the hydrocarbon oil sorbents and cement hydration products. The two locations had very similar elemental compositions, and the only difference between them lay in the concentrations of the elements. On the surface of oil sorbent, carbon, a unique element of the oil sorbent polymer, accounted for the highest percentage of the chemical composition. In contrast, on the cementitious particle, oxygen was the most-detected element, indicating that cement hydration products were in the majority.

**Figure 9:**
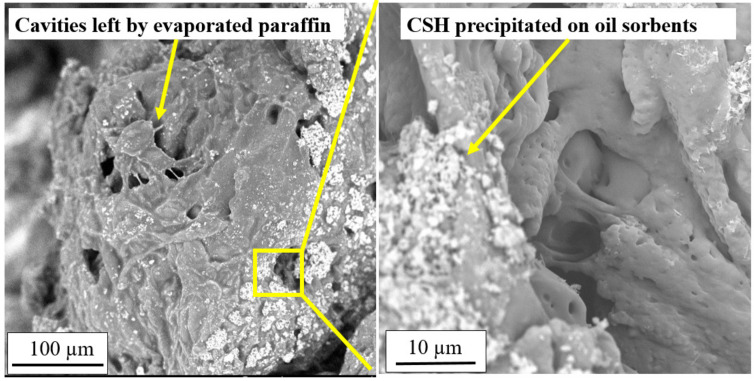
Representative SEM image of swollen oil sorbents in mixed soil samples permeated with liquid paraffin.

After the permeation of liquid paraffin for six hours, the oil sorbent-containing sample was also observed under SEM. Before being sent into the chamber of the SEM, the samples were vacuum dried for 5 days to remove the liquid paraffin from the oil sorbents. The swollen oil sorbent particles were more porous than the unswollen state, and cavities could be seen throughout the particle (Figure 9). These cavities used to be filled with absorbed liquid paraffin, and the evaporated paraffin created the pore-structure in the oil sorbent particle. Despite the permeation of liquid paraffin and swelling of the oil sorbents, cementitious particles were seen to be firmly attached to the oil sorbent polymers. This indicates that the aggressive ingress of organic contaminants had no adverse effects on the bonding between the cement hydration product and the oil sorbents.

### 3.7. Self-Healing Process in Soil Mix Cut-Off Wall Materials Incorporating Oil Sorbents

**Figure 10:**
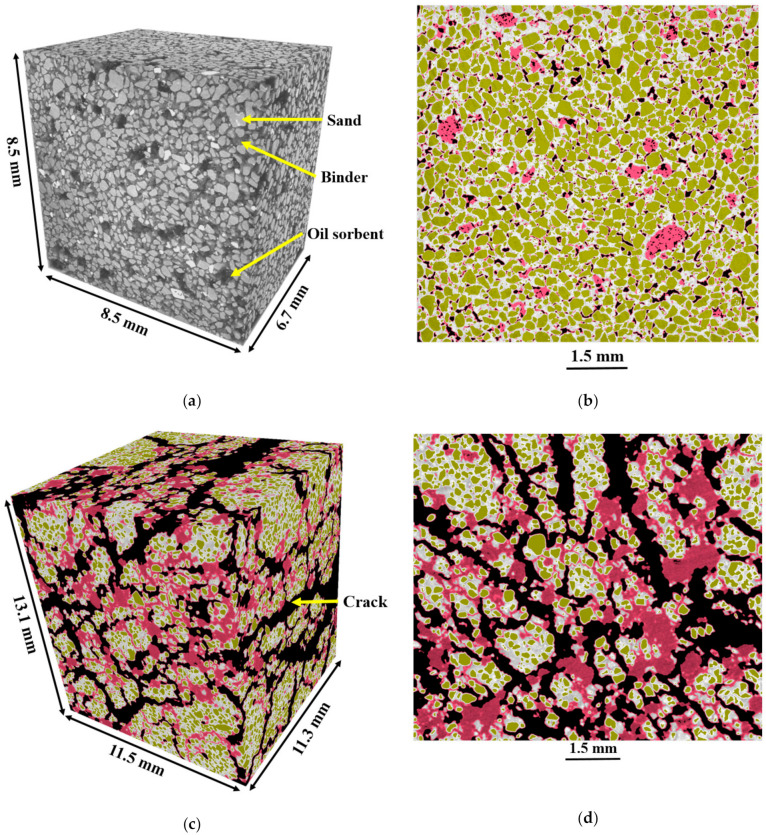
Reconstructed (**a**) 3D and (**b**) 2D image of undamaged OS-10% specimen; (**c**) 3D and (**d**) 2D image of post-healing OS-10% specimen (black for pores and cracks, red for oil sorbents, white for cementitious binders, and brown for sand particles).

Uniform distribution of the oil sorbents in the mixed soil columns is achieved with the laboratory scale auger set-up. This was confirmed with the high-resolution X-ray micro-computed tomography conducted on the undamaged and post-healing mixed soil sample, as shown in Figure 10. The undamaged oil sorbent-containing sample contained four phases: (1) the lightest air phase of the pores within the folded oils sorbent particles and few pores between sand and binder particles that were generated during soil mixing; (2) a light solid phase of oil sorbents; (3) a dense solid phase of cementitious binder, and (4) the densest solid phase of sand particles. For the convenience of analysis, a region of interest, 8.5 mm × 8.5 mm × 6.7 mm, was extracted and the representative 3D and 2D images are shown in Figure 10a,b. The four phases were colored in the 2D image with black for air pores, red for oil sorbents, white for cementitious binders, and brown for sand particles. In the 2D cross-sections, pores were mostly observed within the oil sorbents and not connected with each other.

The post-healing sample was also scanned after the UCS-induced cracking and immersion in liquid paraffin for six hours. Oil sorbents embedded in the cementitious matrix acted as an attachment and binder to prevent the mixed soil sample from crumbling. In addition, it is shown that the swollen oil sorbents with different sizes could effectively block and heal most cracks. In contrast to the vacuum-dried post-healing samples for SEM tests, where paraffin had to be removed from the oil sorbents, the micro-CT scan sample was saturated with liquid paraffin and the CT reflected the microstructure of the swollen oil sorbents more accurately. A region of interest, 13.1 mm × 11.5 mm × 11.3 mm, was extracted and the representative 3D and 2D images are shown in Figure 10c,d. From the 3D image, cracks shown in black with an average width of 525 ± 202 µm were seen randomly distributed over the sample. Without the incorporation of oil sorbents, the propagation of such wide cracks would normally disassemble the mixed soil samples, thus suggesting the benefit of the oil sorbent to protect the integrity of the cracked sample. The polymers absorbed liquid paraffin and swelled to form a saturated solid rubbery mass, so there were no pores within the oil sorbents in the 2D slice, which is an apparent morphological change of oil sorbents triggered by the organic contaminant. These swollen oil sorbents blocked cracks and made it difficult for the permeated liquid to flow into the mixed soil further. In this way, the permeability was reduced once the swelling behavior was triggered and the mixed soil self-healed.

**Figure 11:**
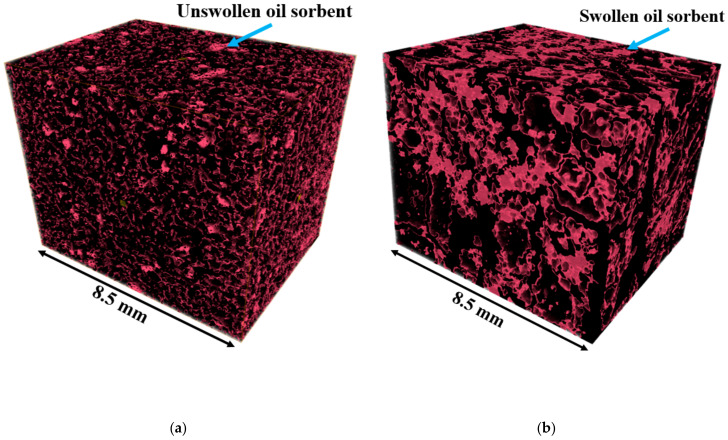
Comparison of the extracted oil sorbent networks of (**a**) undamaged and (**b**) post-healing OS-10% specimen.

To quantify the increase in volume of the swollen oil sorbent, the volumetric fractions of the oil sorbents were measured in the micro-CT analysis. Figure 11 shows the extracted oil sorbent network in the undamaged soil mix sample (left) and post-healing sample saturated with liquid paraffin (right). The volumetric fraction of unswollen oil sorbents was 4.9% by total mix volume, while that of swollen ones was 13.7%. This nearly three-fold increase demonstrates the capacity of the oil sorbent to occupy the air space and block the cracks. In addition, the swollen oil sorbents form large, interconnected polymer networks in the cementitious matrix, which contribute greatly in maintaining the integrity of the mixed soil sample and in preventing the flow of permeated organic contaminants.

## 4. Conclusions

This study developed organic contaminant-triggered self-healing soil mix cut-off wall materials incorporating oil sorbents. The main conclusions were:

1. The laboratory-scale auger mixing produced good quality mixed soil columns with oil sorbent-containing grout. The addition of oil sorbents was found to increase the ductility of the mixed soil and allowed it to deform to a greater extent.
2. The 500 µm-wide cracks could be blocked and the permeability was reduced by an order of magnitude after the permeation of liquid paraffin, indicating that the oil sorbents were capable of absorbing the organic contaminants and swelling to heal the cracks.
3. SEM-EDX tests showed that the oil sorbent particle firmly embedded itself into the cementitious matrix, indicating strong interfacial bonding. Micro-CT scan analysis verified the uniform distribution of the oil sorbents during the mixing process. The oil sorbents swelled three times in volume, triggered by liquid paraffin, and therefore blocked the cracks in the matrix.
4. These promising results exhibit the potential for the oil sorbents to provide soil mix cut-off walls in organically-contaminated land with self-healing properties and enhanced durability.

## Figures and Tables

**Table 1:** The soil-grout mixes used in this study in ratio of the constituents.

Mix	Soil-to-Grout Ratio	Grout (%)				Oil Sorbents (% by Grout Weight)
		PC	GGBS	Bentonite	Water	
Control	4:1	25	25	2.5	47.5	0
OS-5%	4:1	25	25	2.5	47.5	5
OS-10%	4:1	25	25	2.5	47.5	10
